# Development of real-time RT-qPCR assays for the typing of two novel bluetongue virus genotypes derived from sheeppox vaccine

**DOI:** 10.1016/j.jviromet.2021.114288

**Published:** 2021-12

**Authors:** Simon King, John Flannery, Carrie Batten, Paulina Rajko-Nenow

**Affiliations:** The Pirbright Institute, Ash Road, Pirbright, Surrey, GU24 0NF, United Kingdom

**Keywords:** Bluetongue virus, RT-qPCR, Serotyping, Diagnostics

## Abstract

Previously, we reported the detection of two novel bluetongue virus (BTV) strains (SPvvvv/02 and SPvvvv/03), possibly representing new BTV genotypes, in a batch of sheeppox vaccine. We developed type-specific RT-qPCR assays (targeting genome segment 2) for these two new BTV strains. The limit of detection of both assays was 10 genome copies/μl and no cross-reactivity with other BTV genotypes was observed. The performance of three other BTV group-specific diagnostic assays was also tested against the putative novel genotypes. RT-qPCR assays targeting BTV segment 9 and 10 detected both strains (SPvvvv/02 and SPvvvv/03) whereas a BTV segment 1 RT-qPCR assay was unable to detect either BTV strain. The work presented here expands upon the current repertoire of RT-qPCR assays for BTV genotype determination.

## 1 Introduction

Bluetongue (BT) is a disease that affects ruminants such as sheep, goats and cattle which is caused by the bluetongue virus (BTV). It has the potential to cause severe economic losses through animal death, abortion, weight loss and decreased milk production (Rushton and Lyons, 2015) and as such is a world Organization of Animal Health (OIE) notifiable disease. In addition, indirect economic costs are incurred through disease control and prevention strategies which include vector control, vaccination and trade restrictions. For instance, the 2006–2010 northern European BT epizootic incurred estimated costs of €132 M in Germany alone (Gethmann et al., 2020).

BTV is the type species of the genus Orbivirus within the *Reoviridae* family. The BTV genome consists of ten linear segments of dsRNA, which encode seven structural (VP1 to VP7) and five non-structural proteins (NS1, NS2, NS3/3a, NS4, and NS5) (Ratinier et al., 2011; Stewart et al., 2015). The structural protein VP2, encoded by BTV segment 2, is the most variable structural protein and determines BTV serotype. Historically, neutralisation tests – based on the interaction of neutralising antibodies with the VP2 outer capsid protein – have been primarily used to classify BTV into different serotypes. However, in recent years, sequencing of segment-2 has been used for the classification of BTV into genogroup and genotype, rather than serotype. Eleven genogroups (A–K) have been identified so far, each genogroup including several different genotypes/serotypes (BTV-GLUE, http://btv-glue.cvr.gla.ac.uk/#/home). Serotypes BTV-1 to BTV-24 (often termed classical BTV serotypes) are noncontagious and are predominantly transmitted by *Culicoides* biting midges (Breard et al., 2018). In recent years, several novel BTV genotypes/serotypes (often referred to as atypical) have been identified throughout the world such as BTV-25 (Hofmann et al., 2008b), BTV-26 (Maan et al., 2011), BTV-27 (Jenckel et al., 2015; Zientara et al., 2014), BTV-X ITL2015 (Savini et al., 2017), BTV-Y TUN2017 (Lorusso et al., 2018), BTV-Z ITA2017 (Marcacci et al., 2018), BTV-28 (Bumbarov et al., 2020), SPvvvv/02 and SPvvvv/03 (Rajko-Nenow et al., 2020), BTV XJ1407 (Sun et al., 2016), BT 57/08 (Wright, 2014), BTV-MNG1/2018 and BTV-MNG3/2016 (Ries et al., 2020). Some of these atypical BTV serotypes display characteristics not seen in classical BTV serotypes, such as the inability to grow in mammalian or insect cell lines (Chaignat et al., 2009) and the ability to be directly transmitted between infected animals (Batten et al., 2014). The atypical BTV-28 strain was identified in a commercially available sheeppox vaccine (Bumbarov et al., 2016). However, a later study showed that this batch of vaccine was contaminated with not one, but with two novel BTV strains (Rajko-Nenow et al., 2020). Although atypical BTV do not require notification to OIE, a recent study has shown that animals experimentally infected with BTV-28 can exhibit clinical signs of BT and are able to pass the virus to uninfected animals through direct contact (Bumbarov et al., 2020).

BT laboratory diagnosis utilises a group-specific RT-qPCR assay targeting one of the more-conserved regions of BTV genome such as segment 1 (Shaw et al., 2007), segment 9 (Maan et al., 2015) and segment 10 (Hofmann et al., 2008a). As additional BTV serotypes are discovered, it is critical that specific RT-qPCR assays are developed to allow their accurate detection and typing. In this study, we have developed two RT-qPCR assays for the detection of novel ‘putative’ genotypes of BTV: SPvvvv/02 and SPvvvv/03 discovered in a commercial contaminated sheeppox vaccine. For the purposes of this study, we have used the term ‘genotype’ to describe these two novel BTV strains which have been classified according to the sequence of segment 2, in line with the nomenclature proposed by BTV-GLUE (http://btv-glue.cvr.gla.ac.uk/#/home).

## 2 Methods

### 2.1 Design of the RT-qPCR assays

Primers and probes were designed for each putative genotype (SPvvvv/02 and SPvvvv/03) using Primer Express software with default settings. The BTV segment 2 sequence for each genotype (GenBank accession numbers MN723881 and MN723871) was used to design the primers/probe. The sequences of the primers and probes are listed in Table 1.

**Table 1:** Sequences of primers and probes for the typing real-time RT-qPCR assays of SPvvvv/02 and SPvvvv/03. Sequence start and end positions refer to segment 2.

Primer/probe	Sequence	Sequence start	Sequence end
SPvvvv/02_Fwd	5′-CGG TGG ATT GAG CGT TAA-3′	1748	1765
SPvvvv/02_Rev	5′-CGC CCA TGT ACT CGA AAA C-3′	1800	1782
SPvvvv/02_Probe	5′-FAM CAG CAG CAG TCT GC MGB-3′	1767	1780
SPvvvv/03_Fwd	5′-GAC ATC TGC CCA CAT TCG-3′	1838	1855
SPvvvv/03_Rev	5′-GCT TAA CGC GGT TCT TCT G-3′	1894	1876
SPvvvv/03_Probe	5′-FAM TGG CGC GTT ATA CAT T MGB-3′	1858	1873

### 2.2 RNA extraction

RNA was extracted from 100 μL of sample material (virus isolate or PBS-resuspended vaccine) using the LSI MagVet Universal isolation kit (ThermoFisher Scientific, Paisley, UK) and the KingFisher Flex (ThermoFisher). RNA was eluted into 80 μL of MagVet elution buffer and stored at −20 °C until further analysis. MS2 bacteriophage (at a concentration of 10^5^ PFU/ extraction) was used as an internal control (IC) to monitor the virus extraction efficiency and determine the presence of inhibitory substances in the sample matrix.

### 2.3 Virus isolates

The commercial vials of the BTV-contaminated sheeppox vaccine (4 vials from the same batch) were reconstituted in 1 mL Phosphate Buffered Saline (PBS, pH 7.2) and then 500 μL used as inoculum in embryonated chicken eggs (ECE) (Bumbarov et al., 2016), Baby Hamster Kidney (BHK), African green monkey cell lines (Vero), BSR cells (a variation of BHK-21 cells) or KC cells (derived from *Culicoides sonorensis*) as previously described (Oura et al., 2009). Four vials of the BTV-contaminated sheeppox vaccine and virus isolates (n = 26) generated on different cell lines were tested using a suite of RT-qPCR assays for the detection of BTV, including the newly designed type specific RT-qPCRs (SPvvvv/02 and SPvvvv/03).

### 2.4 RT-qPCR

PBS-reconstituted BTV-contaminated vaccine (n = 4), cell culture isolates (n = 26) and BTV-positive samples with unknown serotypes (n = 3) that were submitted previously to the BTV OIE Reference Laboratory at the Pirbright Institute were tested using both SPvvvv/02 and SPvvvv/03 type specific RT-qPCR assays. In addition, BTV group-specific assays targeting: Seg-1 (Shaw et al., 2007), Seg-9 (Maan et al., 2015) and Seg-10 (Hofmann et al., 2008a) were evaluated on the samples.

Extracted RNA was denatured at 95 °C for 5 min prior to RT-qPCR. All BTV RT-qPCR assays were performed on an Applied Biosystems 7500 Fast real-time PCR instrument using the Express One Step SuperScript qRT-PCR kit (ThermoFisher).

Each type specific RT-qPCR was performed in a final volume of 20 μL comprising 1 x reaction mix, 400 nM forward and reverse primers, 100 nM probe, 0.4 μL ROX, 2 μL enzyme and 3 μL of RNA. Cycling conditions were as follows: reverse transcription at 50 °C for 15 min and 95 °C for 20 s, followed by 45 cycles of 95 °C for 3 s and 60 °C for 30 s.

The Seg-1 RT-qPCR assay was performed in a final volume of 20 μL comprising 1 x reaction mix, 800 nM forward and reverse primers, 100 nM probe, 0.4 μL ROX, 2 μL enzyme and 4 μL of RNA. Cycling conditions for the Seg-1 and Seg-9 RT-qPCR assays were the same as the typing assay above. The Seg-9 and Seg-10 RT-qPCR assays were performed with modifications from the published conditions; the final 20 μL reaction comprised 1 x reaction mix, 400 nM forward and reverse primers, 200 nM probe, 0.4 μL ROX, 2 μL enzyme and 5 μL of RNA. The cycling conditions for the Seg-10 RT-qPCR assay were as follows: 50 °C for 15 min, 95 °C for 20 s, followed by 45 cycles of 95 °C for 3 s, 56 °C for 30 s and 72 °C for 30 s.

For the amplification of MS2 IC, the RT-qPCR assay was performed using previously published primers (Wolf et al., 2008). The reaction mix and cycling conditions were the same as for type specific RT-qPCRs, but with 300 nM forward and reverse primers and 200 nM probe. A sample was deemed inhibitory if the IC had a C_T_ ≥3 values higher than the C_T_ of the negative control (molecular biology grade water).

### 2.5 Assessment of specificity, efficiency, and sensitivity of typing RT-qPCR assays

The specificity of the typing RT-qPCR assays was evaluated using RNA extracted from BTV serotypes 1–24, 26, 27 and BT 57/08 along with other viruses, which are either genetically closely related to BTV or cause clinically indistinguishable disease in host species, such as Peste des Petits Ruminants virus (n = 1), Epizootic Haemorrhagic disease virus (n = 1), Sheeppox virus (n = 1), Goatpox virus (n = 1) and Foot-and-mouth disease virus (n = 2).

An ultramer for each of the putative novel BTV genotypes was synthesised by Integrated DNA Technologies (Leuven, Belgium) with its sequence corresponding to 1725−1809bp of SPvvvv/02 segment 2 (MN723871) and to 1827−1912bp of SPvvvv/03 segment 2 (MN723881). In order to determine the efficiency of the RT-qPCR and the limit of detection (LOD), tenfold dilutions (10^7^ to 10 ° copies/μl) of each ultramer were prepared in MagVet elution buffer and each dilution tested in ten replicates. The limit of detection (LOD) was considered as the lowest concentration at which all 10 replicates tested positive. The intra- and inter-assay variability was determined by testing ten replicates of each BTV genotype RNA on three independent RT-qPCR runs. For each typing assay, the percentage coefficient of variation (%CV) was calculated to determine the repeatability of each assay.

### 2.6 Phylogenetic analysis

BTV segment-2 reference sequences were retrieved from GenBank to represent all BTV genotypes that have been proposed by a BTV genome sequence data resource (BTV-GLUE, http://btv-glue.cvr.gla.ac.uk/#/home). Multiple sequence alignment was performed using the ClustalW algorithm of the MEGA7 software (Kumar et al., 2016) and phylogenetic trees were reconstructed using neighbour-joining with the Maximum Composite Likelihood evolutionary distances (Tamura et al., 2004). The reliability of the trees was estimated by the bootstrap test (1000 replicates) and the clade/group was supported at a value ≥70 %.

## 3 Results

### 3.1 Diagnostic sensitivity and specificity

Each of the four reconstituted sheeppox vaccines tested positive using both the SPvvvv/02 (mean C_T_ value 25.64) and SPvvvv/03 (mean C_T_ value 28.08) type specific RT-qPCR assays. Out of 26 virus isolations derived from the commercial vaccine, 5 were positive for both SPvvvv/02 and SPvvvv/03, whereas 21 were positive for one of the two strains (Table 2). All samples were positive by the Seg-10 assay, whereas the Seg-1 assay failed to produce a C_T_ value for any of the samples. A C_T_ value was obtained for 25 samples (4 re-suspended vaccines and 21 virus isolations) using the Seg-9 assay. Samples (n = 4) displaying a C_T_ >32 by the Seg-10 assay were negative by the Seg-9 assay.

**Table 2:** Comparison of BTV group-specific and typing RT-qPCR C_T_ values.

Sample type	Passage history	SPvvvv/02 C_T_ value	SPvvvv/03 C_T_ value	Seg-1 C_T_ value	Seg-9 C_T_ value	Seg-10 C_T_ value
Re-suspended vaccine 1	N/A	24.64	27.09	Undet.	30.88	23.98
Re-suspended vaccine 2	N/A	26.66	29.42	Undet.	31.80	26.14
Re-suspended vaccine 3	N/A	26.77	28.52	Undet.	31.39	25.48
Resuspended vaccine 4	N/A	24.47	27.27	Undet.	28.85	22.99
Cell culture 1	ECE BSR3	Undet.	14.56	Undet.	17.77	13.38
Cell culture 2	ECE BSR2 BHK1	Undet.	11.81	Undet.	16.36	11.43
Cell culture 3	ECE Vero2	Undet.	13.51	Undet.	17.60	13.27
Cell culture 4	ECE	Undet.	11.08	Undet.	14.51	10.42
Cell culture 5	ECE BSR2	Undet.	13.00	Undet.	16.58	12.41
Cell culture 6	BHK2	11.23	36.56	Undet.	14.91	10.34
Cell culture 7	BSR1BHK2	15.80	Undet.	Undet.	20.68	16.40
Cell culture 8	BSR1BHK2	15.94	Undet.	Undet.	19.82	15.68
Cell culture 9	BSR3	16.75	Undet.	Undet.	21.72	16.83
Cell culture 10	BHK2	16.99	Undet.	Undet.	20.65	16.26
Cell culture 11	Vero2	12.51	Undet.	Undet.	17.01	12.31
Cell culture 12	Vero1VeroVDS1	12.70	Undet.	Undet.	17.15	12.51
Cell culture 13	ECE BSR3	12.12	Undet.	Undet.	18.82	11.77
Cell culture 14	ECE Vero2	12.28	Undet.	Undet.	19.16	12.09
Cell culture 15	ECE BSR2BHK1	14.75	Undet.	Undet.	20.94	14.11
Cell culture 16	ECE BSR2	14.45	Undet.	Undet.	22.28	14.06
Cell culture 17	KC1	35.19	36.32	Undet.	Undet.	35.85
Cell culture 18	ECE	13.03	21.82	Undet.	19.04	12.39
Cell culture 19	ECE	16.11	37.22	Undet.	23.47	15.66
Cell culture 20	ECE	Undet.	35.27	Undet.	Undet.	35.70
Cell culture 21	ECE	Undet.	33.07	Undet.	Undet.	32.56
Cell culture 22	ECE	Undet.	10.80	Undet.	13.50	9.09
Cell culture 23	ECE	Undet.	15.67	Undet.	21.30	15.95
Cell culture 24	ECE	Undet.	34.57	Undet.	Undet.	35.47
Cell culture 25	ECE	Undet.	24.22	Undet.	27.07	23.09
Cell culture 26	ECE	24.78	26.65	Undet.	28.85	23.38

The C_T_ values generated by the type specific RT-qPCR assays were correlated with those of the Seg-10 assay (SPvvvv/02 r>0.98, P < 0.0001; SPvvvv/03 r>0.99, P < 0.0001). The mean difference in C_T_ values between the Seg-10 and type specific RT-qPCR assays were not significantly different (0.23, P = 0.09 and 0.44, P = 0.09 for SPvvvv/02 and SPvvvv/03, respectively) (Table 2). The type specific RT-qPCR assays showed strong correlation with the Seg-9 assay; however, this was only significant for the SPvvvv/03 assay (r = 0.9747, P=<0.0001). Despite this correlation, the C_T_ value mean difference between the Seg-9 and type specific RT-qPCR assays was significant (SPvvvv/02 -5.39, P < 0.0001; SPvvvv/03 -3.76, P < 0.0001).

None of the previously untyped BTV samples produced a C_T_ value for either SPvvvv/02 or SPvvvv/03 but were positive using the Seg-10 assay (C_T_ range 29.46–36.90). Neither of the typing RT-qPCR assays produced a C_T_ value for a sample not containing the target serotype RNA.

There was no significant difference (p = 0.19) between the MS2 C_T_ values of the samples (mean C_T_ 26.20, range 25.51–27.24) and the negative controls (mean C_T_ 26.46, range 25.99–27.02), indicating that any potential inhibitory substances were removed during the nucleic extraction procedure.

### 3.2 Efficiency, LOD and %CV of the RT-qPCR

**Fig. 1:**
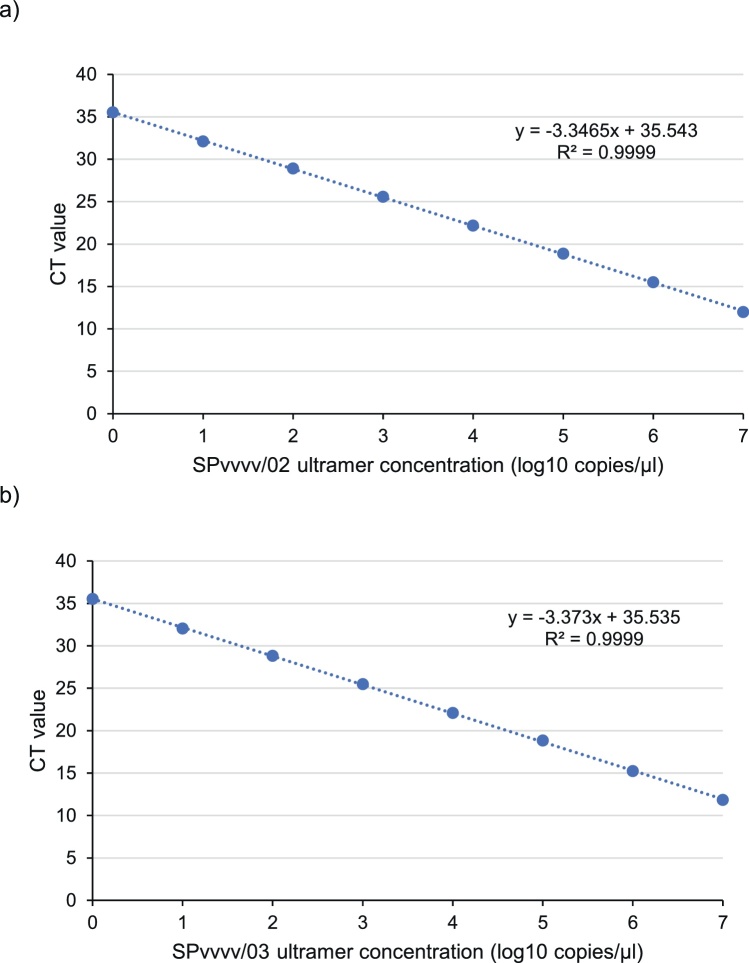
Standard curve for the a) SPvvvv/02 RT-qPCR assay, and b) SPvvvv/03 RT-qPCR assay.

Both type specific RT-qPCR assays displayed a linear relationship between the C_T_ value and the ultramer concentration (R^2^≥0.99) within the range of 10 ° to 10^7^ copies/μl (Fig. 1). The standard curves produced from the dilution series of the ultramers were used to calculate the PCR efficiencies. The PCR efficiencies were 98.98 % and 97.91 % for the SPvvvv/02 and SPvvvv/03 RT-qPCR assays, respectively. These are both within the recommended acceptable range of 90–110 %. The LOD was classified as the lowest concentration of the ultramer for which C_T_ values were determined for all ten replicates. For both RT-qPCR assays, the LOD was determined as 10 copies/μl. The mean intra-assay %CV was 0.84 % and 0.57 % for SPvvvv/02 and SPvvvv/03, respectively, whilst the inter-assay %CV was 1.27 % and 0.73 %. Both assays demonstrated good repeatability and reproducibility, displaying low intra- and inter-assay %CV.

### 3.3 Phylogenetic analysis

**Fig. 2:**
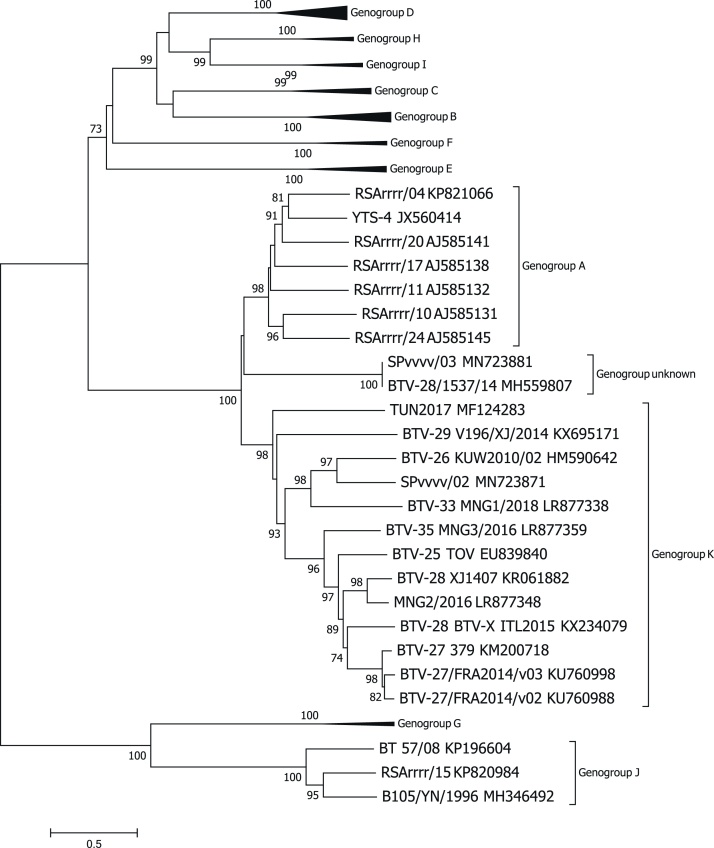
Phylogenetic relationship for the VP2 coding region of BTV. A Neighbour-joining tree (1,000 bootstrap replicates) was constructed using the MEGA7.0 software. Nomenclature used in accordance with that proposed by the BTV-GLUE database.

All segment-2 sequences (n = 35) representing classical BTV genotypes/serotypes were assigned a genogroup (from A to J) and the formation of each genogroup was supported by a bootstrap value ≥98 % (Fig. 2). The majority of atypical BTV strains, including SPvvvv/02, were assigned to genogroup K (supported by a bootstrap value of 98 %). SPvvvv/03 and BTV-28/1537/14 shared a high nucleotide (nt) identity in segment 2 (99.9 %) and clustered together in a separate clade (supported by a bootstrap value of 100 %), possibly representing a new genogroup. The nt sequence identity between SPvvvv/03 and sequences belonging to the genogroup A (60.09–62.57 %) was higher in comparison with the genogroup K (56.59–59.04 %). Ambiguity in BTV nomenclature has been identified for BTV-28 as the following strains; BTV-28/1537/14, BTV-28 XJ1407 and BTV-28 BTV-X ITL2015 were all identified as BTV-28 despite their spread throughout the phylogenetic tree (BTV-GLUE, http://btv-glue.cvr.gla.ac.uk/#/home) (Bumbarov et al., 2020).

## 4 Discussion

Although rare, the unintentional contamination of vaccines, therapeutics and plasma with viruses poses a risk with the potential for devastating consequences (Barone et al., 2020). Sources of viral contaminants are mainly linked to contaminated cell lines, materials and/or reagents used in cell culture and exposure of the cell culture process stream to the environment (Barone et al., 2020). Chinese hamster ovary cells were shown to be contaminated with orbiviruses such as BTV (Burstyn, 1996) or EHDV (Rabenau et al., 1993) in the late 80 s. In the 90 s, a canine distemper vaccine contaminated with BTV-11 resulted in abortion and death of dogs that had been vaccinated during pregnancy (Akita et al., 1994). The contaminated sheeppox vaccine represents a different scenario to the above as the viral contaminant (BTV) is capable of directly causing disease in the intended recipient, in this case sheep or cattle. The use of a contaminated vaccine during a mass vaccination campaign could introduce novel BTV serotypes in naïve populations of sheep (or cattle) with possibly severe consequences. During the vaccine manufacturing process, virus specific PCR assays have been successfully used to identify viral contamination (e.g. before bioreactor harvest) (Barone et al., 2020).

We have developed two type-specific RT-qPCR assays for the detection of two ‘putative’ novel BTV genotypes SPvvvv/02 and SPvvvv/03, originating from the commercial contaminated sheeppox vaccine. Both RT-qPCR assays proved to be highly sensitive and specific enabling typing of BTV positive samples with a low virus load. By using these newly developed typing RT-qPCRs, we confirmed that the same batch of sheeppox vaccine was not contaminated with one novel BTV genotype as previously thought (Bumbarov et al., 2016), but it was contaminated with two novel BTV genotypes (SPvvvv/02 and SPvvvv/03) simultaneously. In addition, we confirmed that both novel BTV genotypes can be detected by the group specific RT-qPCR assays targeting segment-9 and -10 and, for this reason, these assays should be used if BTV-screening is required during vaccine manufacturing. Although the typing assays presented here have been proved to be highly sensitive and specific, their design (as is the case for all RT-qPCR assays) was based upon the limited sequences available during their inception. This may raise concerns as to their ongoing diagnostic sensitivity as new BTV serotypes are sequenced. Thus, there is scope for existing RT-qPCR assays to be assessed on an ongoing basis (Flannery et al., 2019).

Of the group specific RT-qPCR assays tested against the two novel genotypes SPvvvv/02 and SPvvvv/03, the assay targeting segment-10 was the most sensitive. Although the C_T_ values produced by the Seg-10 and typing RT-qPCR assays were comparable, the C_T_ values obtained from the Seg-9 RT-qPCR assay were significantly greater.

The Seg-9 RT-qPCR assay includes two pairs of primers covering the same target region; one specific pair and one degenerate pair (Maan et al., 2015). Through comparison of the SPvvvv/02 and SPvvvv/03 isolate sequences to the Seg-9 RT-qPCR assay primer sequences, it was determined that the assay relies on the degenerate forward primer for the detection of both genotypes, which reduces the efficiency of the PCR and subsequently leads to a loss in sensitivity. As shown by the results in this study, this can lead to false negatives in samples with low levels of virus. The Seg-1 RT-qPCR assay was unable to detect either of the genotypes, irrespective of the samples’ viral load. It has been shown previously that the Seg1 RT-qPCR assay is unable to detect some atypical BTV strains such as BTV-26 (Maan et al., 2011). These results demonstrate the importance of ensuring a diagnostic assay is capable of detecting novel, emerging strains of a virus for successful surveillance and control strategies to be put into place.

The recent advances in high throughput sequencing have led to the discovery of several novel BTV strains, possibly belonging to new serotypes. Although sequencing analysis of segment-2 shows how a novel BTV strain differs from already-known strains in terms of nucleotide sequence identity, this is not sufficient to confirm the identification of a novel serotype. It may not be possible to perform the gold standard virus neutralisation test for the confirmation of serotype if virus propagation fails or antisera against all existing BTV serotypes are not available.

The lack of a standardised approach to genetically describe and classify novel BTV strains has led to inconsistencies in the field of Orbivirus research. Three BTV strains - BTV-28/1537/14, BTV-28 XJ1407 and BTV-28 BTV-X ITL2015 - were all classified as BTV-28 despite differences in phylogenetic clustering. However, BTV-28/1537/14 is very distinct from the remaining two strains and based on our phylogenetic tree could form a distinct genogroup alongside the SPvvvv/03 strain. Interestingly, unlike other atypical BTV strains, BTV-28/1537/14 has been shown to cause clinical signs in experimentally infected ewes (Bumbarov et al., 2020). In addition, numbering of novel serotypes does not seem to be sequential. Considering the above, there is a clear need to standardise BTV classification based on genetic characterisation. Taking these factors into account, we have not proposed a new genotype/serotype number for either of the strains considered in this paper in order to avoid further confusion in BTV nomenclature.

Due to the ever-expanding diversity of BTVs it is important to frequently re-assess the ability of current assays to provide an accurate diagnostic. Prior to this study, there were no type-specific assays for the detection of the putative new genotypes SPvvvv/02 and SPvvvv/03. The work presented in this publication will fill this gap and increases the repertoire of BTV diagnostic RT-qPCR assays.

## Funding

This research was funded by the 10.13039/501100000277Department for Environment, Food and Rural Affairs [grant number SE2622] and Biotechnology and Biological Science Research Council (BBSRC) [through projects BBS/E/I/00007035, BBS/E/I/00007036, and BBS/E/I/00007037]. This publication was also supported by the European Virus Archive goes Global (EVAg) project that has received funding from the European Union’s Horizon 2020 research and innovation program [grant agreement 871029]. The funders had no role in study design, data collection and analysis, decision to publish, or preparation of the manuscript.

## Author statement

Simon King: Validation, Formal analysis, Investigation, Writing – Original Draft, Writing – Review & Editing, Visualization John Flannery: Conceptualization, Resources, Writing – Review & Editing Carrie Batten: Resources, Writing – Review & Editing, Supervision, Funding acquisition Paulina Rajko-Nenow: Conceptualization, Methodology, Formal analysis, Writing – Review & Editing, Visualization, Supervision.

## Declaration of Competing Interest

The authors declare that they have no known competing financial interests or personal relationships that could have appeared to influence the work reported in this paper.
